# Children of Mentally III Parents at Risk Evaluation (COMPARE): Design and Methods of a Randomized Controlled Multicenter Study—Part I

**DOI:** 10.3389/fpsyt.2019.00128

**Published:** 2019-03-26

**Authors:** Hanna Christiansen, Corinna Reck, Anna-Lena Zietlow, Kathleen Otto, Ricarda Steinmayr, Linda Wirthwein, Sarah Weigelt, Rudolf Stark, David D. Ebert, Claudia Buntrock, Johannes Krisam, Christina Klose, Meinhard Kieser, Christina Schwenck

**Affiliations:** ^1^Department of Clinical Child and Adolescent Psychology, Faculty of Psychology, Philipps University Marburg, Marburg, Germany; ^2^Department of Clinical Child and Adolescent Psychology, Faculty of Psychology, Ludwig-Maximilians-University München, Munich, Germany; ^3^Center for Psychosocial Medicine, Institute of Medical Psychology, Heidelberg University Hospital, Heidelberg, Germany; ^4^Department of Social Psychology, Business and Methods, Faculty of Psychology, Philipps University Marburg, Marburg, Germany; ^5^Department of Educational Psychology, Faculty of Education, Psychology, and Sociology, Institute of Psychology, Technical University Dortmund, Dortmund, Germany; ^6^Department of Vision, Visual Impairment & Blindness, Faculty of Rehabilitation Science, Technical University Dortmund, Dortmund, Germany; ^7^Department of Psychotherapy and Systems Neuroscience, Faculty of Psychology and Sports Science, Justus-Liebig-University Gießen, Gießen, Germany; ^8^Department of Clinical Psychology and Psychotherapy, Institute of Psychology, Friedrich-Alexander-University Erlangen-Nürnberg, Erlangen, Germany; ^9^Department of Medical Biometry, Institute of Medical Biometry and Informatics, Heidelberg University, Heidelberg, Germany; ^10^Department of Special Needs Educational and Clinical Child and Adolescent Psychology, Faculty of Psychology and Sports Science, Justus-Liebig-University Gießen, Gießen, Germany

**Keywords:** children of mentally ill parents, transgenerational transmission, mental disorders, intervention, prevention

## Abstract

**Objectives:** Mental disorders are frequent, associated with disability-adjusted life years, societal, and economic costs. Children of parents with a mental illness (COPMI) are at an increased risk to develop disorders themselves. The transgenerational transmission of mental disorders has been conceptualized in a model that takes parental and family factors, the social environment (i.e., school, work, and social support), parent-child-interaction and possible child outcomes into account. The goal of the “Children of Mentally Ill Parents At Risk Evaluation” (COMPARE) study will thus be twofold: (1) to establish the efficacy and cost-effectiveness of a high-quality randomized controlled trial (RCT) with the aim of interrupting the intergenerational transmission of mental disorders in COPMI, (2) to test the components of the trans-generational transmission model of mental disorders.

**Methods:** To implement a randomized controlled trial (RCT: comparison of parental cognitive behavioral therapy/CBT with CBT + Positive Parenting Program) that is flanked by four add-on projects that apply behavioral, psychophysiological, and neuro-imaging methods to examine potential moderators and mediators of risk transmission (projects COMPARE-emotion/-interaction/-work/-school). COMPARE-emotion targets emotion processing and regulation and its impact on the transgenerational disorder transmission; COMPARE-interaction focuses especially on the impact of maternal comorbid diagnoses of depression and anxiety disorders and will concentrate on different pathways of the impact of maternal disorders on socio-emotional and cognitive infant development, such as parent-infant interaction and the infant's stress regulation skills. COMPARE-work analyzes the transmission of strains a person experiences in one area of life to another (i.e., from family to work; spill-over), and how stress and strain are transmitted between individuals (i.e., from parent to child; cross-over). COMPARE-school focuses on the psychosocial adjustment, school performance, and subjective well-being in COPMI compared to an adequate control group of healthy children.

**Results:** This study protocol reports on the interdisciplinary approach of COMPARE testing the model of the transgenerational transmission of mental disorders.

**Conclusion:** The combination of applied basic with clinical research will facilitate the examination of specific risk transmission mechanisms, promotion, dissemination and implementation of results into a highly important but largely neglected field.

**Clinical Trial Registration:** DRKS-ID: DRKS00013516 (German Clinical Trials Register, https://www.drks.de/drks_web/navigate.do?navigationId=trial.HTML&TRIAL_ID=DRKS00013516).

## Introduction

### Children of Mentally Ill Parents

The German social report of the year 2013 indicates a total of 19 million children/adolescents living in 1.6 million single and 8.1 million dual family households (1). Given the estimated lifetime prevalence rate of 27.4% for mental disorders associated with significant disability-adjusted life years (DALYs) for the age group of 18–65 year olds (2), approximately 25% of the children/adolescents in Germany are living with a mentally ill parent, a percentage resembling international rates (3–7). Having a parent with a mental illness has been associated with multiple psychological and developmental risks for children, such as lower academic achievement (8), increased stress-related somatic health conditions (e.g., higher rates of asthma and other atopic diseases (9), internalizing/externalizing symptoms (10, 11), and the development of severe mental illness (SMI) (12). Our own study of *n* = 15.904 adult patients from three different psychiatric/psychosomatic clinics revealed that 65% of the patients had children and that 73.4% of those parents were currently caring for their children (13). The children of the parents of those three different clinics were already exhibiting symptoms of mental disorders themselves (depending on the sample between 15 and 38.4%), thus providing evidence that the trans-generational transmission of mental disorders (TTMD) is a major risk factor for the development of SMI, as demonstrated in numerous other studies (11, 12, 14–16). Long-term studies have shown that children of parents with a mental illness (COPMI) have a higher life-time risk of developing SMI that ranges between 41 and 77%. However, subclinical symptoms often emerge earlier in life (14, 15). The BELLA study revealed a parental mental illness as a powerful risk factor (OR 2.4) for the development of probable mental health problems in children and adolescents (17). Recent studies have added evidence that offspring with two generations previously affected by SMI are at an even greater risk (18, 19). Thus, COPMI are most likely to constitute the next generation of patients with a mental illness (12) associated with significant DALYs and economic costs (20–22). They therefore constitute an essential target high risk group to be addressed by selective prevention programs (23). This is the aim of the COMPARE-family project (see part II of the study protocol published in this research topic by Stracke et al. (24).

### Transgenerational Transmission

A recent meta-analysis of the trans-generational transmission of parental mental disorders on children's symptomatology revealed specific effects of parental disorders on children (12). According to this meta-analysis, both transgenerational concordance (specific parental mental disorders increase children's risk for certain disorders) and multifinality (parental mental disorders increase children's risk for mental disorders in general) are used to define the scope of the child's diagnostic outcomes of a particular parental disorder. The concepts of transgenerational specificity and equifinality, respectively, refer to similarities and differences in parental disorders preceding a child's diagnostic outcome. Multi- and equifinality are more common in children of parents with unipolar and bipolar affective disorders, whereas the risk of children of anxious parents is mainly restricted to develop anxiety disorders. For all children, risk transmission is assumed to be partly specific since studies indicate a strong tendency for children to develop the same disorders as their parents. A meta-analysis (*k* = 61 studies included) of the cross-sectional association between paternal and maternal psychopathology on children's internalizing and externalizing behavior problems further revealed parent-specific gender effects (25). There is a general lack of studies on the specific risk transmissions for the broad range of parental disorders other than affective and anxiety disorders (12) and studies assessing a wider range of parental disorders as well as their effects on the children are missing, as are studies on parental comorbidity. This lack is addressed in the current study, as we will consider a broad range of parental disorders as well as comorbid ones.

**Figure 1:**
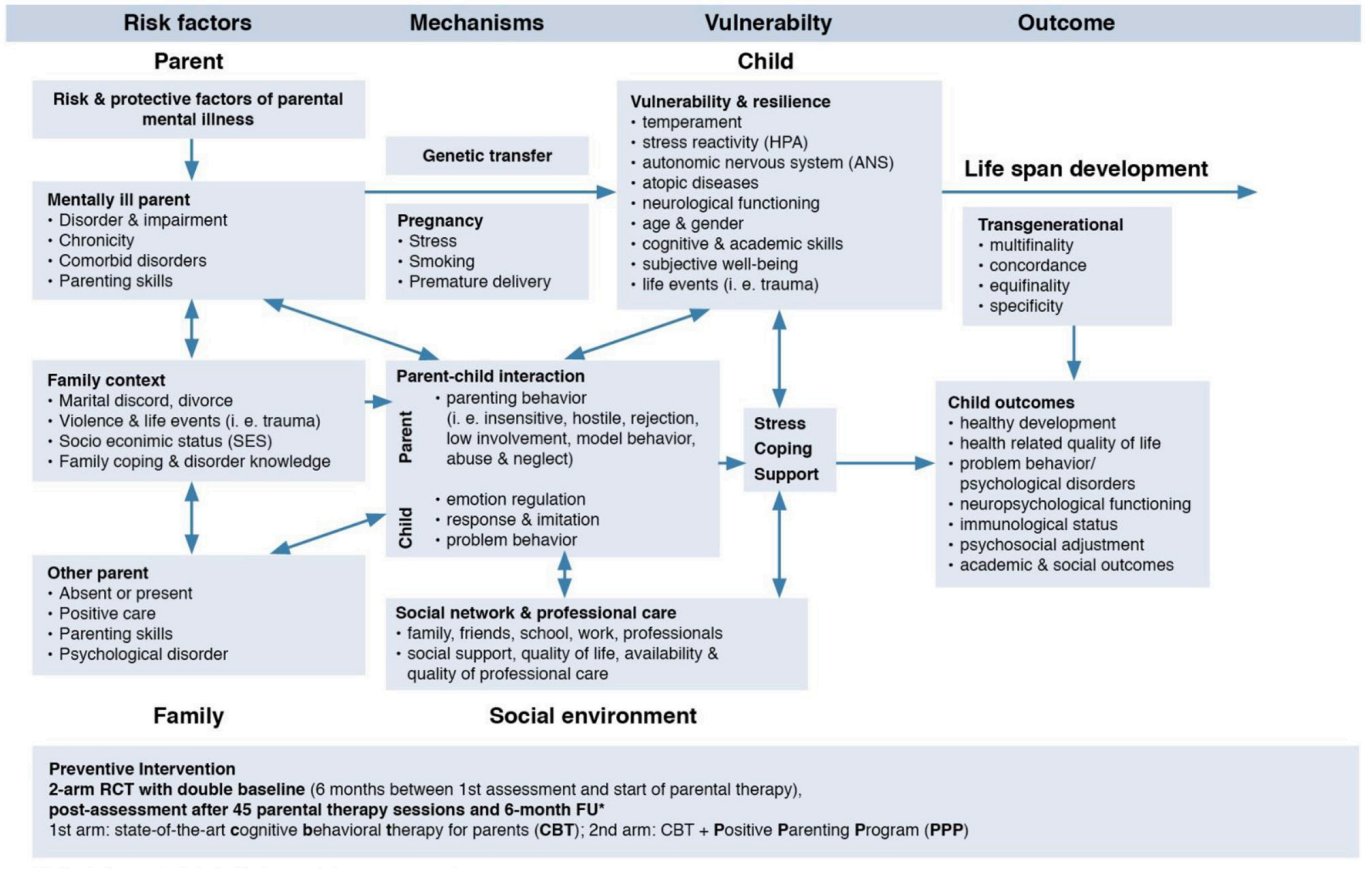
Model of the trans-generational transmission of mental disorders adapted from Hosman et al. (14), p. 253, identifying risk factors, mechanisms, vulnerability, and child outcome. The influence of interventions on the different aspects of the model is an inclusion of the consortium.

A comprehensive model (see Figure 1) of the TTMD identifies four major domains [(1) parent, (2) family, (3) child, and (4) social environment] that interact with their respective systems and are influenced by five transmission mechanisms [(1) genetics, (2) prenatal factors, (3) parent-child-interaction, (4) family, and (5) social factors]. Child development over its whole span is considered, as well as the concepts of multi- and equifinality, concordance, and specificity. While there is empirical support for the model's different domains and factors, many research has solely addressed single dimensions (i.e., focusing on family or child factors or on genetics), without taking the whole model into account, which is clearly necessary (for a review of the model components see (13). Furthermore, most of those studies were conducted on individual disorders in parents, and comparative studies on the range of parental disorders and on crucial factors such as parental comorbidities are rare (26) or entirely lacking (12). Moreover, the specific impact of the various moderating and mediating processes between the incidence of mental disorders in parents and their children—such as genetics, epigenetics, stress reactivity, emotion regulation, parenting skills, parent-child-interaction, children's cognitive abilities, family, and environmental influences—as well as their interactions have not been clarified so far. Thus, the aim of the COMPARE consortium with its subprojects COMPARE-family/emotion/interaction/school/work is to test the TTMD model and to simultaneously assess the four major domains as well as the transmission mechanisms. This will enable us to establish specific transmission profiles (equi- vs. multifinality vs. concordance vs. specificity) for a range of parental disorders with/without comorbidities. Furthermore, we will be able to identify risk-profiles for children at high vs. low risk, since studies have shown that not all children will develop disorders themselves (25) although their overall risk is significantly increased (14). This will improve the development of targeted interventions, connecting our first aim with the second one, since the bi-directional influences of interventions on the TTMD as well as specifics of the TTMD on interventions have not been investigated so far.

Summing up, we expect that our study will substantially deepen our knowledge of the relevant risk transmission mechanisms and related child outcomes, as well as markedly improve children's health, by simultaneously assessing the domains and mechanisms of the TTMD model as well as the bi-directional influences of a selective intervention in one study. This paper reports on part I of the overall protocol paper describing the consortium structure with the clinical trial and the four add-on research projects. Part II is the protocol paper of the clinical trial, also published in this research topic (24).

### Aims of the Research Consortium

The research consortium “Children Of Mentally III Parents At Risk Evaluation” (COMPARE) is a network of researchers that includes disciplines from clinical, work and organizational, developmental, and educational psychology. Using a randomized controlled trial (RCT) and accompanying add-on projects, the COMPARE consortium addresses the following main goals:

1. To establish effects of parental treatment on child outcome [see the protocol on COMPARE-family by Stracke et al. (24) in this Frontiers research topic].
2. To tests the domains of the TTMD model within the RCT.
3. To identify specific transmission mechanisms.

The primary hypotheses of the model testing part of the study are:

COMPARE-family: To test the transgenerational transmission of mental disorders from parents to children. We will establish risk profiles and analyze the transmission pathways of multifinality, equifinality, specificity, and concordance. We further test the effects of parental psychotherapy treatment on the children and whether a training of parenting skills will result in additional effects [for details see (24) in this research topic].

COMPARE-emotion: Emotion processing and emotion regulation demonstrate processes that, on the one hand, are reduced in COPMI compared to children of parents without mental illness. This relationship should be moderated by factors related to the mental diseases of the parents, such as the type of disorder, disease duration, age of the children at disease onset and disease severity. On the other hand, we assume these processes to be influenced by parental treatment and to simultaneously moderate the effectiveness of the intervention. Therefore, the current subproject was designed to (a) compare COPMI with children of parents without mental illness (COPWI) regarding emotion processing (EP) and emotion regulation (ER) skills, (b) to evaluate the impact of EP and ER on treatment outcome, and (c) to assess the effects of different treatments on EP and ER in COPMI.

COMPARE-interaction: Maternal psychopathology, infant stress reactivity, and the quality of the parent-infant interaction at 3–4 months predict infant development at 24 months postpartum, and maternal comorbid depression and anxiety coincide with greater impairment in the infant development at 12, 18, and 24 months than depression alone. Furthermore, it is hypothesized that the parent-infant interaction as well as the infant stress reactivity at 12 months of age mediate the relationship between the maternal psychiatric diagnosis 3–4 months postpartum and infant development (socio-emotional as well as cognitive) at 24 months of age.

COMPARE-work: We expect to observe that mentally ill parents are confronted with more severe working conditions as compared to healthy parents, and that stronger adverse spillover effects of work-based strain toward the family emerge for them. With respect to the transmission of parents' strain to their children, we as well assume that such crossover-effects depend on the parents' health status. Overall, we expect to identify specific patterns with regard to the spillover-crossover effect assuming that the maladaptive cycle leading from the parents' poor working conditions (in particular those with a mental illness) via spillover to the family can be interrupted so that no crossover to their children occurs.

COMPARE-school: We expect that COPMI perform poorer on academic achievement scales, display less positive psychosocial adjustment patterns, and suffer from lower general and domain-specific subjective well-being compared to children of mentally healthy parents. Due to the specific parental mental disease and parents' gender, we anticipate identifying different effects in the aforementioned child outcome variables in COPMI.

## Methods

### COMPARE-Family: Effects of Parental Psychotherapy and Additional Parent Training on Children

This subproject is central for the entire consortium and planned as a randomized controlled trial establishing the effects of high quality parental therapy (cognitive behavioral therapy/CBT) on the children in comparison to parental CBT and a parent training, the Positive Parenting Program (CBT+PPP). We have chosen CBT for the intervention, as CBT has the soundest evidence base for the psychotherapeutic treatment of mental disorders (72). Studies further demonstrate poorer parenting skills in parents with mental disorders (27–30), and the enhancement of such skills has been a significant mediator in improving child outcomes (31). The Positive Parenting Program (PPP) is a well-established program to enhance parenting skills, and specific effects on child psychopathology could be demonstrated (32, 33). Studies explicitly testing effects of parental treatment in conjunction with parenting skills are lacking so far.

A total of seven university based study sites participates in the RCT and each of them specializes on different mental disorders. We will thus be able not only to address depression and anxiety, but a wider range of disorders such as sleep-wake-, and somatic symptom disorders, obsessive compulsive, trauma and stressor related disorders, eating and personality disorders, bipolar, and schizophrenic disorders. As patients in Germany are required to be abstinent for outpatient psychotherapy, patients with substance use disorders will not be included in the trial.

The structure of the RCT will allow us further (i) to establish effects of the parental disorder(s) with/without comorbidities on children's health; (ii) to establish effects of the different interventions (CBT vs. CBT+PPP) on children's health; (iii) to test assumptions of the TTMD model and bi-directional influences of different treatments on the model; (iv) to analyze specific transmission mechanisms; and (v) to establish risk profiles for children with urgent needs for preventive interventions.

Key inclusion criteria are parents with a mental illness in outpatient treatment caring for a child between 1.5 and 16 years of age. Key exclusion criteria are insufficient German language skills, severe impairment of the children requiring comprehensive treatment.

#### Description of the Primary Efficacy/Test Accuracy Analysis and Population

The primary analysis is conducted according to the intention-to-treat (ITT) principle and includes all randomized patients. The confirmatory test for treatment group differences between T2 and T3, and T2 and T4 with respect to the primary endpoint each applies linear mixed multi-level model with patients at level 1 and children at level 2, adjusting for center, number of comorbidities, number of children, baseline TRF score at T2, and length of waiting period between T1 and T2 (in weeks). The overall type I error rate is set at 5% (two-sided) and will be controlled by applying the multiple test procedure for hierarchically ordered hypotheses. Effect size assumed for estimation of sample size is set at *d* = 0.25. With a two-sided significance level of α = 0.05 and a power of 1–β = 0.8 using a two-sample *t*-test, 253 patients per group (n = 506 total) are required. Taking a drop-out rate of 20% into account, *n* = 634 patients need to be enrolled into the trial. Since it is assumed that parts of the outcome variance can be explained by the inclusion of covariates, the actual power of the analysis by linear mixed multi-level model is expected to be higher than 1–β = 0.8. Sample size calculation was performed using SAS v9.4. For details on COMPARE-family and the full study protocol please refer to Stracke et al. (24) in this research topic.

The projects COMPARE-emotion/interaction/work/school are closely linked to this trial, as the parents of children of COMPARE-family will also be assessed for those subprojects. We will thus gain knowledge on the interaction of therapy effects with the specified TTMD-domains.

### COMPARE-Emotion: Emotion Processing and Regulation in Children of Parents With Mental Illness

The TTMD model identifies child factors as one of four major domains of the transmission of parental mental disorders to the child (see above). Therefore, specific characteristics and behavioral traits in the child are likely to raise or lower the risk for a mental disorder in the child of a parent with a mental illness for him- or herself. Emotion processing and emotion regulation have been identified as such factors. Emotion processing (EP) is a broad concept that includes sub-processes of emotion perception, affective stimulus interpretation, and affective reaction to another's emotions. In the current sub-project, we want to focus on (a) emotion recognition, (b) perspective taking, and (c) affective arousal (sympathy vs. personal distress) as a reaction to another person's emotional situation (34). EP aberrations constitute a central characteristic of both individual and a wide range of mental disorders, reflecting a transdiagnostic approach (35–37). Emotion regulation (ER) comprises processes applied by individuals to influence the incidence, kind, intensity, and duration of their emotions as well as their effects on feelings and behaviors (38, 39). Strategies can be adaptive, for example if they increase positive or decrease negative emotions, or be maladaptive, having the opposed effect. Both, EP and ER constitute important skills that are transdiagnostically related to mental disorders. Both skills are affected by parental behavior throughout child development. The research conducted on this topic so far reveals direct and indirect associations between a parental mental illness and aberrant EP or ER in their children. However, no comprehensive study on the impact of the child's EP and ER on the trans-diagnostic trans-generational transmission of mental disorders has been conducted so far, despite the fact that these processes constitute important targets for preventive interventions. Furthermore, the impact of EP and ER on treatment effects has not been studied to date.

**Figure 2:**
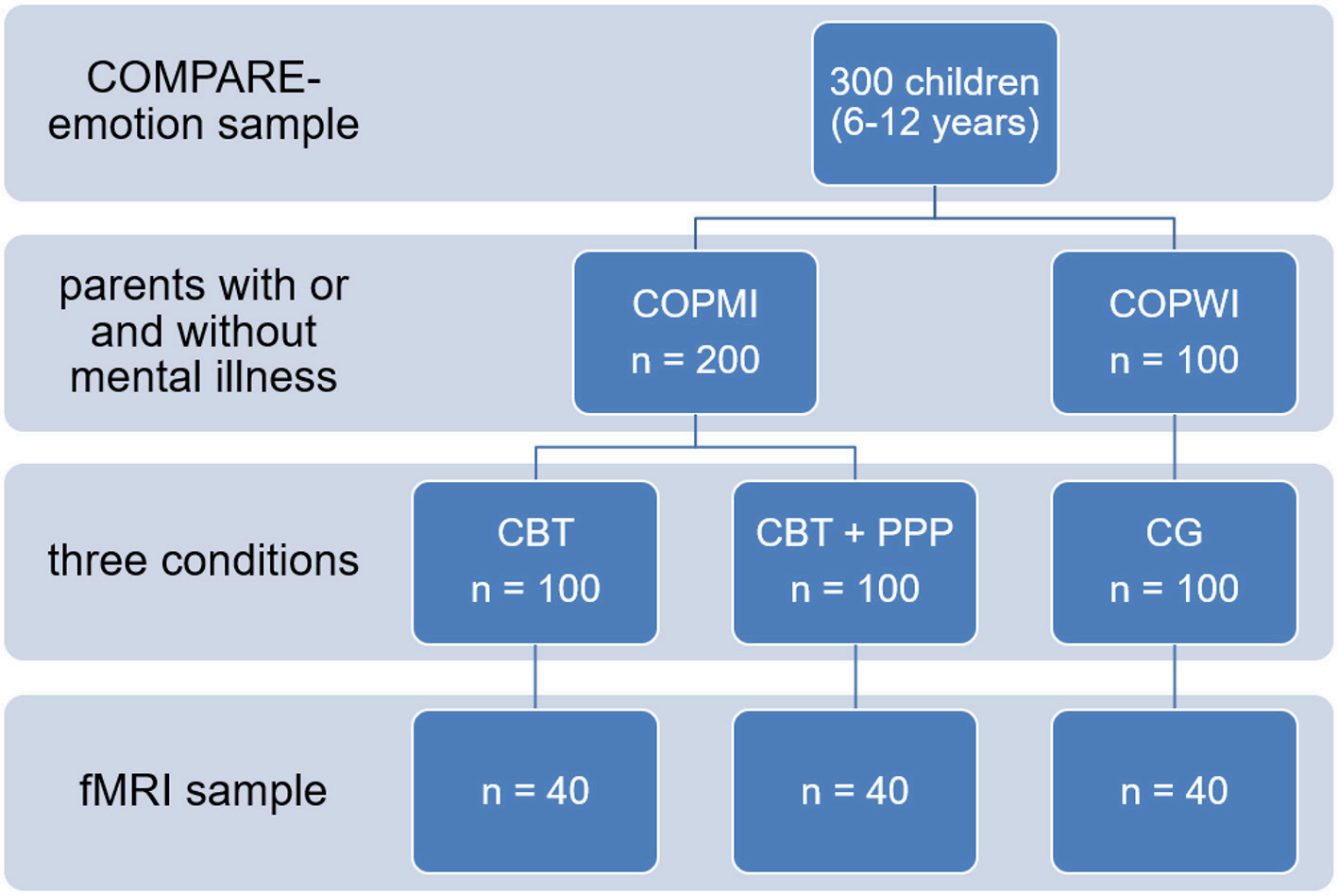
Sample of COMPARE-emotion. fMRI, functional magnetic resonance imaging.

To examine these processes, experimental tasks assessing EP and ER will be administered in 200 children of parents participating in the COMPARE-family study [see study by Stracke et al. (24) in this research topic] at the assessment points T1, T3, and T4 of the clinical trial (100 children of parents receiving cognitive behavioral therapy and 100 children of patients additionally receiving the Positive Parenting Program). Furthermore, a sample of 100 children with parents without a history of mental diseases or psychotherapy and no indication of current mental disorders will be assessed with the same tasks at a single measurement point (see Figure 2). A version of the Video Sequences Task will measure various aspects of emotion processing including emotion recognition, perspective taking, personal distress, and sympathy. This task has been chosen because of it's high ecological validity. Children are shown short film clips containing scenes with the protagonist conducting a certain basic emotion. Additionally, the morphing task will be adopted to assess emotion recognition with respect to quality and speed. In this task, film clips with neutral faces morphing into affective faces are shown and participants are supposed to press a button as soon as they recognize the emotion. To measure emotion regulation, an emotional Go/Nogo paradigm will be applied. In this task children are supposed to press a button following certain affective stimuli, e.g., happy faces, and inhibit a button press following other stimuli, e.g., sad faces. These two experimental tasks allow for a precise and fine grained assessment of the relevant constructs. Dependent variables include behavioral task performance measures and peripheral physiological activity during the tasks (heart rate variability, skin conductance, and mimicry). Furthermore, questionaires will measure emotion regulation strategies and cognitive and emotional empathy of the children and their parents.

To investigate differences in emotion processing between COPMI and COPWI not manifest in behavioral or physiological measures, patterns of neuronal activity and neuroanatomical connectivity will be compared between subgroups. Especially the amygdala-prefrontal cortex network is assumed to display a specific coordinating role in this context, and different mental disorders in childhood and adolescence have been found to be associated with aberrant amygdala and/or prefrontal cortex activation during EP. Therefore, out of each subgroup 40 participants will be measured with functional magnetic resonance imaging. A paradigm will be applied that involves processing of emotional faces as well as implicit emotion regulation via emotion labeling. We assume that COPMI will show higher activity in emotion processing brain areas and less habituation of the amygdala than COPWI, in particular if there is no task to perform. When the participants are required to draw attention away from the facial emotional expressions, we additionally expect higher neural costs in the COPMI, especially overactivity in the anterior cingulate cortex. During emotion labeling, COPMI should exhibit less regulatory prefrontal activity than COPWI, i.e., less activity of the ventrolateral prefrontal cortex combined with higher activity in the amygdala and less functional connectivity between both structures. Additional diffusion tensor imaging will be applied to investigate differences in white matter tract integrity in emotion processing networks.

### COMPARE-Interaction: Impact of Maternal Comorbid Depression and Anxiety in the Peripartum Period on Infant Development: The Role of Parent-Infant Interaction and Infant Stress Reactivity

The Compare-interaction subproject pursues a developmental approach which aims to identify risk factors for the generational transmission of parental mental health disorders in early childhood as displayed in the TTMD model. This model identifies parent-child interaction as a core mechanism in the transgenerational transmission of parental mental disorders to the child. COMPARE-interaction focuses on the long-term consequences of maternal depressive and comorbid depressive and anxiety disorders during the peripartum period on child development, especially on socio-emotional and cognitive development. Special attention will be paid to the mediational effect of parent-infant interaction and infant stress reactivity on the relationship between the maternal disorder (depressive, comorbid, healthy controls) and infant outcome. Moreover, it will significantly contribute to model testing in the model's following domains: mentally ill parent, family context, other parent, parent-infant interaction, infant outcome. Given the high prevalence of depressive and anxious disorders during the peripartum period and the increased risk for children of depressed and anxious mothers to exhibit adverse developmental problems, further research in this field is urgently needed. The negative impact of depression on infant socio-emotional and cognitive development is well-documented in the literature (40). To date, there are no studies comparing mothers with peripartum depression alone to depressed mothers with comorbid anxiety disorders. As comorbidity is associated with greater impairment and symptom severity related to the primary diagnosis, comorbidity in mothers might raise their offspring's risk of developing internalized disorders even more than has been noted in conjunction with depression alone (41).

N = 168 families (n = 56 per subgroup and n = 84 per study center in Heidelberg and Munich) will be recruited through inpatient and outpatient centers as well as maternity hospitals in Munich and Heidelberg. A drop-out rate of 20% is expected.

**Figure 3:**
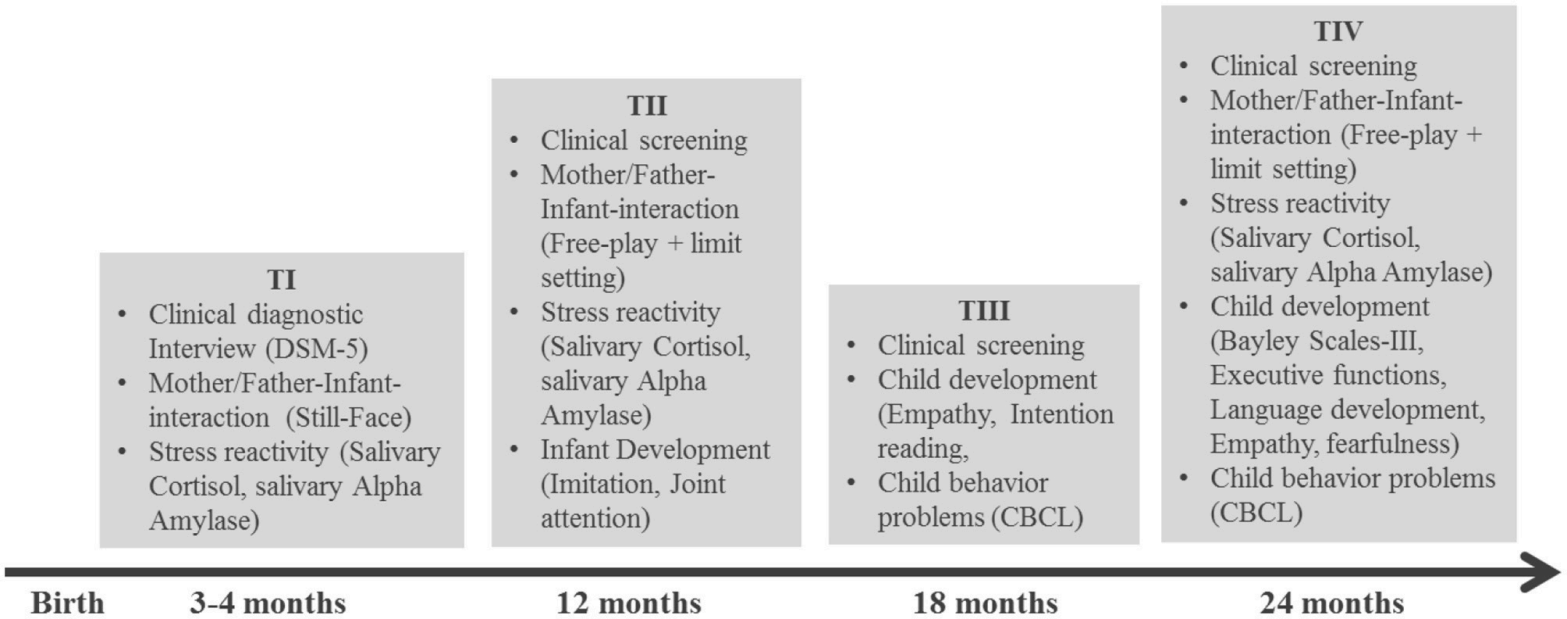
COMPARE-interaction study design.

This study is designed to assess peripartum depressed mothers with and without comorbid anxiety disorders according to DSM-5, fathers and their infants, as well as a healthy control group at four measurement points over the first 2 years (T1: 3–4 months postpartum, T2: 12 months postpartum, T3: 18 months postpartum, an T4: 24 months postpartum; see figure 3 for details). Besides the evaluation of parental psychiatric status at all measurement points, parent-infant interaction will be videotaped and coded according to the Coding Infant Behavior Scales (CIB) (42). To determine infant stress-reactivity, cortisol will be extracted from infant saliva, which is collected before (C1), 20 min (C2), and 30 min after the interactional episodes.

At the age of 12 months, declarative and imperative point production and understanding (43), as well as imitation of object-related (44) and intransitive action skills (45) as predictors of later social-cognitive development will be assessed. This will be expanded upon at 18 months by an assessment of empathy (46) and intention reading (47) as milestones of constructive social behavior and mindreading, and at 24 months of empathy, executive functioning (gift delay (48) and reverse categorization (49), language abilities (SET-K (50), and child fearfulness [spider task (51)]. The measurements taken at 24 months will serve as our primary outcome measures for the longitudinal study. Infant socio-emotional development will be rated by mothers, fathers and additional caregivers via the Child Behavior Checklist/Caregiver Teacher Report Form (52, 53) at T3 and T4. Cognitive development will be assessed at T4 using the Bayley Scales of Infant Development-III (54).

This sub-project will yield new insights into the specific effects of maternal depressive and comorbid depressive and anxiety disorders disorders on different aspects of the infant's social and cognitive development, as well as on the interaction mechanisms contributing to this process. We need a deeper understanding of the underlying mediation effects to develop future prevention or intervention approaches, since research on comorbid depressive and anxiety disorders and their impacts on the parent-infant relationship and infant development is still lacking. Therefore, better understanding will enable us to suggest starting points for further research into this area. The multi-perspective approach is novel as we will be analyzing socio-emotional and cognitive parameters together and also include fathers in this study. This is unique as no other research projects have studied these research questions using additional biopsychological measures. Research of this kind is particularly important to help us to better understand adverse developmental pathways and to pave the way for future implementation of prevention and intervention programs.

### COMPARE-Work: Parental Work Environment as a Risk Factor: Spillover-Crossover Effects From Parents to Children

Work has a central meaning to our lives: it shapes our identities, offers us social support and appreciation, and helps us to achieve collective goals (55). How important work is and the effects it has on children can be illustrated by the example of being unemployed. Studies indicate that the children of unemployed parents are disadvantaged: their birth weight is lower, they reveal slower growth and have more accidents (56, 57), they exhibit more self-destructive behavior, and that they tend to drop out of school and are more frequently unemployed as adults [“intergenerational unemployment”; (57)]. Similar effects are to be expected if working conditions are detrimental (58); then long-term negative health-effects occur that can spread over to the family and thus be a risk factor for the mental health of children. A negative downward spiral could be activated when parents, because of being emotionally exhausted for example, cannot provide their children any support when doing the homework while their children absorb their parents' work-related worries. Parents' adverse working conditions may have a negative impact on both their children's school performance and mental well-being. To the best of our knowledge, no research so far has attempted to clarify this process.

A first aim of this project is to explore whether mentally ill as compared to healthy parents are confronted with more severe working conditions, namely that they are confronted with more stressors (e.g., time pressure), possess fewer resources (e.g., lack of social support) at their workplaces, and report on a worse career development (e.g., job loss). Across countries findings of the (59), p. 30) revealed that the “average income of people with a moderate mental disorder is around 90% of the total working-age population, and it is 80% or less (…) for those with a severe mental disorder.” These statistics suggests that also mentally ill parents face a high unemployment risk or work more frequently in precarious jobs. Moreover, research has identified two different ways in which work demands or strain are carried over: spillover and crossover. Adverse working conditions can strain parents, and such strain is expected to be transferred from the work domain toward the family, rendering it a risk factor for family life (work-family conflicts) and hence for the mental health of children. This “spillover” is the within-person, across-domain transmission of strain from one area of life to another (60). The next step following spillover-processes from work to family is to explore parent-to-child crossover, which has not been studied to date. Crossover represents a phenomenon, where experiences of stress and strain are transmitted between individuals (61). The core assumption is that one's own stress and strain experiences have an impact on others in close surroundings. To clarify the underlying mechanism in the spillover-crossover-process (62), we will try to shed light on the mediators. As proposed in the concept of emotional contagion (63), negative states are transferred easily and automatically. Moreover, crossover can be of cognitive nature through shared social cognition, a factor that can be a source of information to facilitate the interpretation of ambiguous situations. Hence, the way crossover from parents to children works—driven emotionally or cognitively—will also be researched.

In this project, working conditions of parents of children of the RCT [COMPARE-family; see (24) in this research topic] and parents of a representative group of school children not differing in basic demographic variables (assessed together with COMPARE-school) will be compared. We will assess task- and job-related working conditions (e.g., time pressure, autonomy, interruptions at work, and skill utilization) with the Instrument for Stress Oriented Task Analysis [ISTA; (64)] and the Copenhagen Psychosocial Questionnaire [COPSOQ II; (65)]. Further, working conditions on the social level (e.g., social stressors, social support, appreciation) will be explored using well-established measures. To explore spillover, besides working conditions, strain (work-family-conflict, need for recovery) and well-being (life satisfaction, mood, emotional exhaustion) of both groups of parents will be assessed via existing self-report measures. To study crossover, also the children will be investigated regarding their well-being (life satisfaction, mood, emotional exhaustion) and their closeness to their parents. To detect the underyling transmission process, an *ad-hoc* measure that reflects emotional vs. cognitive processes in crossover for children will be developed and pre-tested.

Using the software G-Power and specifying alpha as 5% and statistical power to equal at least 80% and applying MANOVA to test for differences between mentally ill vs. healthy parents' working conditions considering parents' gender, a minimum sample size of 200 parents is suggested. However, to test the more complex models (SEM) detecting the spillover-crossover process, a minimum sample size of 300 parents would be optimal to detect medium-sized effects. As higher attrition rates are not uncommon for longitudinal research in the Occupational Health Psychology field, we plan to contact a total of 887 mothers and/or fathers. Depending on the parents' consent to participate—which we expect to be about 50% (aiming at 450 parents in total)—the exact sample size will be somewhat lower, though.

In sum, this project will deepen our knowledge of the TTMD model. By assessing parental working conditions, an essential element in the TTMD model can be explained. Various stressors and resources in the working context of both mentally ill and healthy parents as well as work-related strain and work-family conflict indicators will be explored, characterizing the aforementioned spillover-crossover process in (all) its complexity.

### COMPARE-School: Psychosocial Adjustment, School Performance, and Subjective Well-Being in Children of Mentally Ill Parents

Most studies have focused on the mental health and child psychopathology of COPMI and just a few studies have focused on other relevant child outcomes proposed in the TTMD, such as academic attainment, psychosocial functioning or subjective well-being by investigating an adequate control sample of children with healthy parents [e.g., (66–68)]. One main aim of this subproject is to compare a school control sample to the COMPARE-family intervention groups [see (24) in this research topic]. This control sample will be recruited in elementary and secondary schools during regular school lessons. School children comparable in age to the RCT [COMPARE-family; see (24) in this research topic] will be examined with the same tests as COPMI. All tests will be given during regular school lessons to entire school classes. About *N* = 900 school children will initially be investigated. Within this sample, a propensity score matching [see (73)] will be performed controlling for age, gender, and parental socioeconomic background to obtain a school control cohort comparable to and as large as the COPMI group.

Different indicators of academic achievement will be assessed (standardized achievement tests in reading and mathematics, school grades). Social skills will be measured via self-reports as well as via parental assessments. Subjective well-being will be examined by investigating both cognitive as well as affective variables regarding life as a whole as well as regarding family, school, and peers (69).

Moreover, there is a serious lack of knowledge how parental mental disease affect lower educational attainment. Besides parenting behaviors that are related to child learning (such as homework support) and that in turn affect academic achievement, other mediating child characteristics might also be of influence (e.g., temperament and cognitive abilities). In this context, the child's school values and academic expectations are especially interesting, as research has demonstrated that these variables both differ between children of mentally ill and healthy parents and influence academic achievement (70, 71). Besides other child outcomes such as psychosocial functioning and subjective well-being, especially parenting skills and parenting styles in general might be important process variables.

Research has shown that some COPMI reveal no detrimental outcomes regarding their educational attainment, psychosocial adjustment, or subjective well-being [e.g., (6)]. There is little research to date comparing those children with children suffering from their parent's mental health disorder to identify protective and risk factors. The TTMD model describes various potentially-relevant vulnerability factors such as temperament, cognitive skills or the parenting style and parenting behavior. These vulnerability factors will be additionally examined within COMPARE-school.

Finally, treatment-related changes in educational attainment, psychosocial functioning, and subjective well-being will be examined. The aforementioned outcomes will be assessed at T2 (2nd baseline), T3 (post-test), and T4 (6 months follow-up) in the cohort of COPMI and in the representative control group [for a full description of the RCT-design see (24) in this research topic].

The outcome variables measured in COMPARE-school are closely related to children's health, and provide a holistic perspective onto a child's healthy development. Our project will yield important insights into the specific transmission profiles proposed in the TTMD. In sum, we will contribute to both model testing and the intervention emphasis of the main COMPARE project.

## Transfer into the Routine Provider System

The results of the COMPARE consortium will be made available to the larger scientific community via peer-reviewed publications in scientific journals, presentations at scientific meetings and presentation on the COMPARE website. Partners will be encouraged to publish in “Open Access” journals whenever appropriate, ensuring accessibility to the widest readership worldwide. Throughout the study and beyond, patients, parents, and carers (stakeholder board) will be kept informed about the study through the COMPARE website, flyers, newsletters, and personal contacts. Politicians, public health services, and stakeholders will be informed via teaching seminars and conferences on the background and results of the present study, thus improving public policy and health care decisions whose aim it is to prevent and treat COPMI. Two large health care insurances, the “Allgemeine Ortskrankenkasse/AOK” and “IKK Südwest” have ensured us to disseminate results to their insurance holders. Existing networks (i.e., Kindernetzwerk Deutschland, Diakonisches Werk Baden, unith e. V., BVKJ, DGPS, DGKJP, DGPPN, DGKJ) will be used to disseminate our results to relevant target groups. A joined patient conference on parenting skills is planned. Since all participating Psychology Departments have outpatient departments for children/adolescents and adults and are members of unith e. V. (a collaboration among university-based therapeutic training institutions), the implementation of results in ongoing and future practice is warranted, especially since many of the researchers involved already teach at the respective therapy training institutes and unith e. V. supports this project. The consortium's members also include experts from child and adolescent psychiatry as well as pediatrics, meaning our results will be implemented in those relevant fields as well. As we will be cooperating with a large adult psychiatry institution having many clinics throughout Germany (Schön-Kliniken), our results are certain to be implemented within a major stakeholder. Results will be relevant for and thus included in treatment guidelines as well as in psychology textbooks. Furthermore, as we have included experts from educational psychology will help transfer our findings to educational practice.

## Discussion and Limitations

COMPARE with its subprojects family, emotion, interaction, work, and school, is the first comprehensive research programme on COMPI in Germany, as to date there is neither standard care nor systematic research with respect to this highly vulnerable group.

The COMPARE-family project is the central RCT testing the effects of high quality parental CBT on the children and whether additional PPP will result in incremental effects above and beyond CBT alone [for details see (24) in this research topic]. Effects of this trial as well as baseline data is informative for the other subprojects, as based on this trial the different domains of the TTMD (influences of emotion processing, parent-child-interaction environmental influences such as work and school) can be tested. So far the TTMD model has not been comprehensively tested.

The COMPARE-emotion subproject focuses on emotion processing and emotion regulation as potential child factors for the transgenerational transmission of mental disorders. Furthermore, these processes will be assessed as predictors and outcome measures for the RCT. Emotion processing and emotion regulation will be assessed with the help of different approaches, comprising behavioral measures, peripheral physiological markers, and neuro-imaging techniques. Thus, we expect to accomplish a comprehensive understanding of relevant child factors that display a significant role in the TTMD model. Moreover, the identification of underlying processes is equivalent to the identification of targets for effective intervention in the future.

The COMPARE-interaction subproject focuses on the long-term consequences of maternal psychopathology during the peripartum period on infant development, especially on socio-emotional and cognitive development. Special attention will be paid to the mediational effect of parent-infant interaction and infant stress reactivity on the relationship between the maternal disorder (depressive, comorbid, healthy controls) and infant outcome. Moreover, it will significantly contribute to the model testing in the model's following domains: mentally ill parent, family context, other parent, parent-infant interaction, infant outcome. This sub-project will yield new insights into the specific effects of maternal mental disorders on different aspects of the infant's social and cognitive development, as well as on the interaction mechanisms contributing to this process. It is of great importance to gain a better understanding of the underlying mediation effects to develop future prevention or intervention approaches, since research on comorbid depressive and anxiety disorders and their impacts on the parent-infant relationship and infant development is still lacking.

COMPARE-work explores the working conditions of mentally ill parents in contrast to those of healthy parents, identifies how these working conditions impact on work-related strain and how this causes strain in family life (work-to-family spillover). The comparison of strain and well-being levels in both groups of parents as well as their children enables us to detect transmission effects (parents-to-children crossover), thereby focusing on the specific transmission mechanisms (mediators), and finally analyzes whether treatment buffers or aggravates the spillover-crossover process (moderators). With respect to the preventive intervention, the consideration of parental work-environments is a significant moderator of therapy outcome. More or less supportive or adverse work-environments can be seen as framing conditions (partly outside the control of a person) shaping the opportunities of a therapeutic intervention at least partly; in particular in such cases where working conditions cause, strengthen or work to maintain mental diseases.

COMPARE-school focuses on different indicators of academic achievement, social functioning, and subjective well-being. A main aim is to compare the children of mentally ill parents with a healthy school control sample in terms of academic and psychosocial outputs and to seek variables that explain those differences. Moreover, we will test whether the specific type of parental mental illness and the mentally ill parent's gender are relevant concerning the aforementioned child outcomes. To create targeted interventions for children suffering from their parent's mental disease, we will additionally focus on those children with a low risk for developing health problems themselves and examine how they differ from those who have mental health problems. This comparison will enable us to explore approaches that can support children suffering from their parent's mental disease. Treatment-related factors in academic and psychosocial outcomes will be examined as well. Taken together, we will examine the theoretical model underlying COMPARE in terms of different outcomes other than psychopathology in COPMI and we will test for intervention effects by taking another adequate control sample into account.

However, there are also some limitations to be considered.

First of all, due to funding decisions, some aspects of the TTMD-model, such as (epi-) genetics and somatic outcomes will not be part of the study. However, if this study proves to be successful in the identification of risk mechanisms, this might provide a strong basis for follow-up (epi-)genetic projects on this topic.

Second, the research programme with a clinical trial at the center [see (24) in this research topic] and the four add-on projects puts high demands on the participating patients and their families that are already under increased stress due to the parental mental illness. On the other hand, the intensive assessment of different areas of life that might be impaired or prove to be associated with resources will substantially increase our knowledge on when to provide which interventions in what areas of life for whom.

## Ethics Statement

The study is carried out according to the Good Clinical Practice (GCP) guidelines, the Declaration of Helsinki and its later supplements and local legal requirements. The lead ethics committee at the department of psychology at Philipps-University Marburg approved the study procedure and all study documents. A positive ethics committee vote is required at a study site, before the inclusion of a first patient at the respective site.

## Dissemination

Via peer-reviewed publications in scientific journals, the results of this study will be made available to the scientific community. Using PsychData all primary data will be made available for re- and meta-analyses. Politicians, public health services and stakeholders will be informed throughout the study and beyond, thus, improving public policy and health care decisions concerning preventive interventions and treatments for COPMI.

## Author Contributions

HC has drafted the main manuscript. CR and A-LZ wrote the section on COMPARE-interaction and commented on the whole manuscript. KO wrote the COMPARE-work section and commented on the whole manuscript. RS and LW wrote the COMPARE-school section and commented on the whole manuscript. SW, RS, and CS wrote the COMPARE-emotion section and commented on the whole manuscript. DE and CB are the study advisories, read the manuscript, and commented. JK, CK, and MK are the trial biometricians, read, and commented on the manuscript.

## Members of the Consortium and Recruiting Centers

COMPARE-family (P1): Study coordination Marburg: Hanna Christiansen, Kristin Gilbert, Winfried Rief, Markus Stracke. Data management and biometry Heidelberg: Meinhard Kieser, Christina Klose, Johannes Krisam, Moritz Pohl. Data monitoring Erlangen: Claudia Buntrock, David Daniel Ebert. Recruiting centers: Bochum: Sören Friedrich, Jürgen Margraf, Silvia Schneider; Bielefeld: Angelika Schlarb, Anna-Lena Zurmühlen; Leipzig: Julian Schmitz, Cornelia Exner; Gießen: Pauline Leiders, Arleta Luczejko, Christina Schwenck, Rudolf Stark, Julia Zimmermann; Landau: Jens Heider, Katharina Köck, Annette Schröder. München: Mitho Müller, Corinna Reck, Christian Woll.

COMPARE-emotion (P2): Gießen: Pauline Leiders, Arleta Luczejko, Lena Krüger, Christina Schwenck, Rudolf Stark; Dortmund: Sarah Weigelt, Klara Hagelweide.

COMPARE-interaction (P3): Heidelberg: Nora Nonnenmacher, Anna-Lena Zietlow, Sabine Herpertz; München: Mitho Müller, Corinna Reck, Christian Woll.

COMPARE-work (P7): Marburg: Benjamin Frank, Nathalie Lenninger, Kathleen Otto.

COMPARE-school (P8): Dortmund: Ricarda Steinmayr, Linda Wirthwein.

### Conflict of Interest Statement

The authors declare that the research was conducted in the absence of any commercial or financial relationships that could be construed as a potential conflict of interest.
